# Research on cardiovascular functioning in minority groups: From biological explanations to social and discrimination-related determinants

**DOI:** 10.1016/j.puhip.2026.100826

**Published:** 2026-07-08

**Authors:** Alessandro Carollo, Seraphina Fong, Cristina Ottaviani, Roberto Baiocco, Fiorenzo Laghi, Gianluca Esposito

**Affiliations:** a Department of Psychology and Cognitive Science, University of Trento, Corso Bettini 84, Rovereto, 38068, Italy; b Department of Psychology, Sapienza University of Rome, Via dei Marsi, 78, Rome, 00185, Italy; c IRCCS Santa Lucia Foundation, Via Ardeatina, 306/354, Rome, 00179, Italy; d Department of Developmental and Social Psychology, Sapienza University of Rome, Via dei Marsi, 78, Rome, 00185, Italy

**Keywords:** Autonomic functioning, Minority stress, Stigma, Sexual and gender minorities, Ethnic minorities, Neurological minorities

## Abstract

**Objectives:**

Minority groups consistently exhibit poorer health outcomes than majority populations, a pattern often attributed to chronic exposure to distal and proximal stigma-related stressors. Such sustained stress is thought to disrupt autonomic regulation, and reduced heart rate variability is frequently cited as an indicator of physiological dysregulation associated with heightened vulnerability to adverse health outcomes.

**Study design:**

The present work implemented a scientometric approach to review the scientific literature on the physiological functioning of minority groups (e.g., racial, ethnic, gender, sexual, and neurological minorities).

**Methods:**

A total of 1945 papers published between 1953 and 2024 were retrieved from Scopus. Using CiteSpace, we analysed co-citation patterns and identified nine main research themes, along with the ten most impactful publications in the field.

**Results:**

The bibliometric results revealed a predominant contribution from authors based in the United States, whose work has largely focused on cardiometabolic disparities among ethnic minority populations. Additionally, we observed a clear shift in the research trajectory: from early efforts attributing ethnic health disparities to genetic and dietary differences, towards more recent investigations emphasising the deleterious effects of discrimination on minority health.

**Conclusions:**

This evidence map highlights the need to include discrimination, stigma, and minority stress in cardiovascular and autonomic health research, while improving evidence on sexual, gender, and neurodivergent minorities to guide public health priorities and prevention strategies.

**What this study adds**

• This scientometric review maps the intellectual structure and historical evolution of research on cardiovascular and autonomic functioning in minority populations.
• The findings identify a shift from earlier biological and lifestyle explanations of minority health disparities towards research emphasising discrimination, racism, minority stress, and social environments.
• The study highlights important evidence gaps, particularly the limited attention devoted to sexual, gender, and neurodivergent minorities in cardiovascular and autonomic health research.

**Implications for policy and practice**

• Public health policies addressing cardiovascular disparities in minority populations should consider discrimination, stigma, racism, and minority stress as relevant social determinants of physiological health.
• Surveillance systems and research funding priorities should promote the systematic inclusion of under-represented groups, particularly sexual, gender, and neurodivergent minorities.
• Clinical and public health practice should move beyond individual-level risk factors and account for chronic social stressors and structural barriers when assessing and addressing minority health disparities.

## 1 Introduction

In health care research, individuals belonging to minority groups tend to report poorer health compared with their peers [1]. Studies report that ethnic minorities have higher morbidity and mortality rates for several diseases [1,2]. Similarly, lesbian, gay, and bisexual (LGBTQ+) individuals have been found to experience mental and physical health disparities when compared to heterosexual and cisgender individuals [3,4].

The higher morbidity and mortality risk in minority groups have been interpreted in several ways. For example, genetic factors, socioeconomic level, and migration-related habits or lifestyle changes have been suggested as reasons for ethnic minority and migrant populations’ susceptibility to cardiovascular disease [5]. However, socioeconomic factors do not entirely explain disparities in the incidence, treatments, or outcomes of adverse cardiovascular events in this population [1,6].

One widely accepted explanation for health disparities among minorities is that individuals belonging to minority groups are consistently exposed to and confront challenging social situations, which, in turn, contribute to poorer health outcomes [7,8]. Compared to their counterparts in majority groups, minorities often encounter discrimination, face unequal socioeconomic status, and have limited access to health care [9]. According to the minority stress theory [10,11], these social experiences induce a state of chronic stress that, over time, can result in deteriorating health [12]. Contributors to minority stress include increased rates of discrimination at familial, social, cultural, and workplace levels [13,14]. In addition to these external stressors, individuals from minority groups may also experience proximal stressors, such as internalised negative feelings about their minority identity [11]. Elevated psychosocial stress has been linked to physiological dysregulation and hypertension [15]. Among physiological indicators, autonomic nervous system functioning assessed through heart rate variability has been widely associated with cardiovascular and psychological health as well as mortality risk. The so-called cardiovascular conundrum, a paradoxical pattern characterised by increased sympathetic vasoconstriction alongside elevated vagally mediated heart rate variability, has been observed in ethnic, gender, and sexual minorities (e.g., Refs. [16,17]) and is thought to reflect chronic regulation of emotional responses to discrimination. Overall, minority stress theory suggests that distal and proximal stressors produce sustained psychosocial stress that, over time, contributes to autonomic dysregulation.

Understanding the impact of challenging social experiences (e.g., discrimination) on the health of minority groups is important and socially relevant. The present work aims to investigate the literature on autonomic functioning in the cardiovascular system among minority populations through a scientometric methodology as applied in previous studies (e.g., Ref. [18]). In a data-driven manner, the scientometric approach analyses large samples of literature and their accompanying co-citation patterns to identify the most impactful publications and the main themes in the literature. By systematically mapping the existing research, this study provides a comprehensive overview of how autonomic nervous system functioning has been studied in minority populations, identifying gaps in knowledge and potential directions for future research.

## 2 Methods

### 2.1 Data collection

The methodology of this scientometric study utilises the standardised procedure implemented in existing scientometric literature (e.g., Ref. [18]).

Data were retrieved on 28 February 2024 from Scopus using the search string TITLE-ABS (( “heart rate variability” OR “heart rate” OR “HRV” OR “parasympathetic” OR “autonomic” OR “vagal” OR “vagus” OR “cardiovascular” OR “hypertension”) AND (“prejudice” OR “racism” OR “sexism” OR “sexual minorit*” OR “ethnic minorit*” OR “racial minorit*” OR “gender minorit*” OR “sexual orientation” OR “minoriti*ed” OR “asian minorit*” OR “black minorit*” OR “minority group*” OR “neurodiscriminat*” OR “neuro-discriminat*”) ).

Scopus was used as the data source due to its extensive coverage of indexed journals and documents. A total of 1945 publications published between the years 1953 and 2024 were downloaded and their 93,703 references were retrieved. The 1945 retrieved publications were subsequently analysed using the *bibliometrix* package on R to identify the most involved countries, the most productive authors, the major journals, and the most occurring keywords [19]. The results of this bibliometric analysis are presented in the Supplementary Materials.

### 2.2 Data import

In order to implement a scientometric analysis, the retrieved publications and corresponding references were imported into the CiteSpace software (CiteSpace 6.3.R1 (64-bit) advanced) [20]. The 93.74% of the references (88,020 out of the total 93,703 references) were deemed valid by CiteSpace. When importing data into CiteSpace, only references that include all essential bibliometric elements in the correct format – author, year of publication, title, source, volume, pages, and DOI – are considered valid by the software.

### 2.3 Document co-citation analysis

A document co-citation analysis (DCA) was conducted through CiteSpace to identify primary research domains and main publications within the literature on the physiological functioning of minorities. DCA examines how frequently research papers are cited together (i.e., co-cited) by other publications [21]. The co-citation frequency indicates shared thematic domains and research trends within a specific field [20]. To investigate the patterns of co-citations, DCA creates a network. Each citing and cited document is represented as a network node, co-citations among documents are represented as network links, and the frequency of co-citation is represented as link weight [22].

### 2.4 Network optimisation

**Fig. 1:**
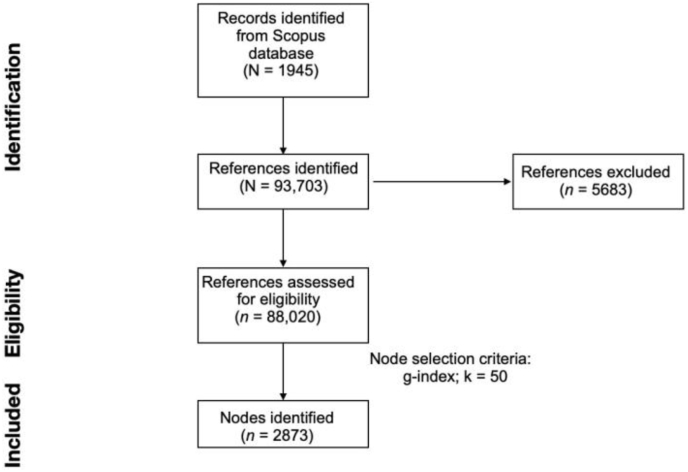
Flowchart outlining the process of literature search, evaluation steps, and network generation.

A balanced DCA network is achieved by setting the selection criteria options (g-index, Top N, and Top N%) in CiteSpace to appropriate values. The DCA network parameters of the present work were optimised according to previous scientometric literature (e.g., Ref. [18]). G-index with *k* set at 50 was found to be the optimal criterion for the present work as it showed the best structural properties (e.g., modularity, silhouette, number of clusters). Fig. 1 summarises the methodological steps of the study, from the data collection to the final number of nodes included in the DCA network.

### 2.5 Network metrics

The evaluation of the generated DCA networks is done using three structural and two temporal metrics. The first structural metric is modularity Q, which measures how well a network can be divided into individual clusters [23]. The second structural metric is the silhouette score, which measures the internal consistency of a cluster and its level of separation from neighbouring clusters within the network [24]. The last structural metric is betweenness centrality, which measures the degree to which a node acts as a bridge between two other random nodes [25].

The first temporal metric is citation burstiness, which uses Kleinberg's algorithm to measure a publication's sudden increase in publications over time [26]. The second temporal metric is sigma, which measures the scientific novelty and impact of a publication by combining its importance within the network (centrality) and its initial impact (burstiness), i.e., (*centrality* + 1)^*burstiness*^ [23].

## 3 Results

### 3.1 Document co-citation analysis

**Fig. 2:**
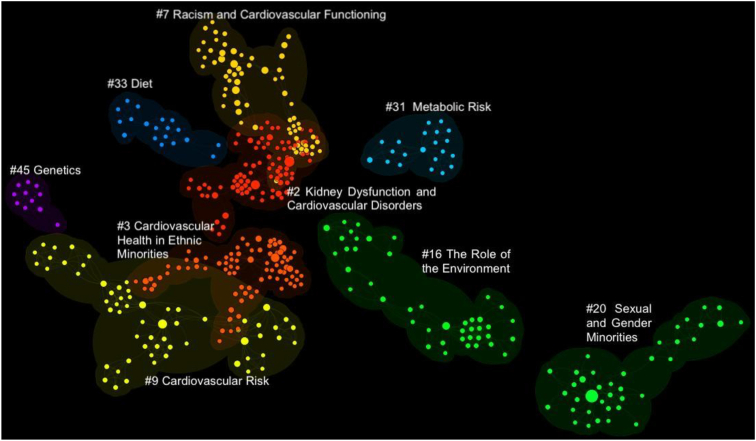
Document co-citation analysis (DCA) network of the literature on physiological functioning in minority groups. The image was generated with the CiteSpace software [20].

The DCA resulted in a network of 2873 nodes and 7636 links (see Fig. 2). The network's modularity score was 0.9643 and its weighted mean silhouette score was 0.9897. These scores suggest that the resultant network had a high degree of modularity, characterised by consistent and clear clusters around specific themes.

Nine major thematic clusters emerged in the network (see Table 1). The top three largest clusters were cluster #2 “Kidney Dysfunction and Cardiovascular Disorders” (size = 86; silhouette = 0.986; mean publication year = 2001), cluster #3 “Cardiovascular Health in Ethnic Minorities” (size = 85; silhouette = 0.986; mean publication year = 2007), and cluster #7 “Racism and Cardiovascular Functioning” (size = 68; silhouette = 0.983; mean publication year = 2011). Each cluster was labelled automatically by the Log-Likelihood Ratio (LLR) algorithm in CiteSpace. The authors then manually reviewed the automatically generated labels by inspecting the main citing papers and the main cited papers within each cluster. Suggested labels were assigned when necessary to better reflect the thematic content of these documents and the overall conceptual coherence of the cluster.

**Table 1:** Information regarding the cluster ID, size, silhouette, mean year of publication, log-likelihood ratio label, and suggested label of the nine major clusters identified with the document co-citation analysis (DCA). The Log-Likelihood Ratio (LLR) label was automatically generated by the software.

ID	Size	Silhouette	Mean Year	LLR Label	Suggested Label
2	86	0.986	2001	expert consensus document	kidney dysfunction and cardiovascular disorders
3	85	0.986	2007	cardiovascular health research	cardiovascular health in ethnic minorities
7	68	0.983	2011	arterial stiffness	racism and cardiovascular functioning
9	63	0.999	2000	black youth	cardiovascular risk
16	43	0.988	2007	social inequality	the role of the environment
20	39	1.000	2009	population-based study	sexual and gender minorities
31	21	1.000	2000	obesity ethnicity	metabolic risk
33	19	0.991	1998	lactose intolerance	diet
45	11	0.983	1997	genetic bottleneck	genetics

Ten unique documents with a citation burst were identified. The document with the highest impact was a report examining the state of the research (including research gaps and future directions) pertaining to the LGBTQ + community across the lifespan (Garofalo et al. [27]; citation burst = 8.50 starting from 2013 and to 2019). The subsequent two most impactful documents were authored by Carnethon et al. [6] (citation burst = 8.18 from 2021 to 2024) and Churchwell et al. [28] (citation burst = 7.55 from 2021 to 2024).

## 4 Discussion

A scientometric analysis was conducted to identify the main trends and influential publications on the physiological functioning of minority populations. Nine major thematic clusters emerged. The clusters are discussed in chronological order based on the mean publication year of their associated documents. Key citing papers are reported using two metrics: coverage (number of cited documents referenced) and global citing score (GCS; total citations in Scopus).

### 4.1 Cluster #45: Genetics

Cluster #45 was the earliest cluster in the literature on physiological functioning in minorities (average year of publication = 1997). The only major citing document was by Carlos Poston et al. [29] (coverage = 10; GCS = 28), which examined genetic and psychosocial predictors of hypertension in US-born African Americans and African-born immigrants. The study found that age, body mass index, and birthplace were associated with hypertension risk, whereas genotype distributions were not.

Similarly, cited documents in this cluster explored genetic and lifestyle predictors of hypertension across racial and ethnic groups (e.g., Ref. [30]). This cluster reflects an early research focus on genetic and biological explanations for cardiovascular disparities, although findings increasingly pointed to the relevance of sociocultural and environmental factors.

### 4.2 Cluster #33: Diet

The subsequent cluster was Cluster #33 (mean year = 1998). The only major citing document was by Jarvis and Miller [31] (coverage = 12; GCS = 85), which examined the prevalence and health implications of lactose intolerance in minority populations (e.g., African Americans, Hispanics, and Asians). The study suggested that low intake of calcium and dairy-related nutrients may contribute to chronic conditions disproportionately affecting these groups.

Similarly, cited documents focused on the role of dietary practices and nutritional factors in cardio-metabolic health disparities (e.g., Ref. [32]).

### 4.3 Cluster #9: Cardiovascular risk

Both clusters #9 and #31 had the year 2000 as their average year of publication. Cluster #9 (size = 63) will be discussed first as it was bigger than cluster #31 (size = 21).

The major citing document in Cluster #9 was by Wyatt et al. [33] (coverage = 10; GCS = 219), which examined the relationship between racism and cardiovascular disease risk in African Americans, considering institutional, interpersonal, and internalised forms of racism [34]. The findings suggested a positive, though not entirely consistent, association between experiences of racism and cardiovascular health risk.

Similarly, studies in this cluster examined the impact of racial discrimination on cardiovascular health among ethnic minority populations, including African American [35] and Hispanic communities [36]. This cluster marks an important shift in the literature towards linking experiences of discrimination and stigma with cardiovascular health disparities.

### 4.4 Cluster #31: Metabolic risk

The major citing document in Cluster #31 was by See et al. [37] (coverage = 8; GCS = 20), which reviewed the health implications of obesity, with particular attention to obstructive sleep apnoea in the United States. The authors highlighted that racial and ethnic minorities are disproportionately affected by obesity-related morbidity and mortality due to factors such as genetic predisposition and socioeconomic status.

Similarly, studies in this cluster examined obesity prevalence and mortality, the influence of socioeconomic and ethnic factors on treatment outcomes, and the relationship between obesity and sleep apnoea [38,39]. This cluster highlights early research linking minority health disparities to obesity-related metabolic and respiratory risks.

### 4.5 Cluster #2: Kidney dysfunction and cardiovascular disorders

The next cluster was Cluster #2 (mean year = 2001). Major citing documents included the ones authored by Greenland et al. [40] (coverage = 15; GCS = 470) and Lakkis and Weir [41] (coverage = 13; GCS = 10). These studies examined disparities in kidney and cardiovascular functioning among ethnic minorities. Evidence suggested that ethnic minority groups face higher risks of kidney disease due to the greater prevalence of cardiovascular and metabolic conditions, as well as barriers to accessing high-quality health care [41,42].

Cluster #2 shows that, following earlier work focused on cardiovascular and metabolic dysfunctions in minority populations, the literature began to highlight an additional area of vulnerability: kidney health disparities.

### 4.6 Cluster #3: cardiovascular health in ethnic minorities

Clusters #3 (size = 85) and #16's (size = 43) average year of publication was 2007. Cluster #3 will be discussed first as it is bigger.

The major citing document of Cluster #3 was by Agyemang et al. [5] (coverage = 13; GCS = 51), which highlighted the persistent uncertainty surrounding the causes of cardiovascular disease disparities among ethnic minority and migrant populations. The authors proposed a framework comparing minority groups across host countries, similarly developed nations, and countries of origin to better identify relevant risk factors.

Similarly, studies in this cluster examined cardiovascular health among ethnic minorities across different contexts (e.g., Refs. [43,44]). Rather than relying solely on broad minority–majority comparisons, these studies increasingly emphasised the importance of study designs capable of capturing contextual complexity, including migration history, sociocultural background, and country-of-origin effects. This line of work underscored that cardiovascular disparities can only be accurately interpreted when these factors are explicitly considered, promoting the development of more refined and comparative epidemiological approaches in minority health research.

### 4.7 Cluster #16: The role of the environment

Cluster #16's major citing document was by Gravlee [45] (coverage = 14; GCS = 484), which argued that health disparities between racial groups cannot be explained by genetics alone, but also reflect social and environmental influences such as systemic racism. Other studies in this cluster examined the role of race, discrimination, and socioeconomic inequalities in shaping health outcomes (e.g., Ref. [46]). This clusters marks an important expansion of focus, highlighting that biological differences alone cannot account for observed disparities without considering the broader sociocultural environment in which minority populations live.

### 4.8 Cluster #20: Sexual and gender minorities

The major citing documents in cluster #20 were authored by Lick et al. [4] (coverage = 9; GCS = 531), by Bosse et al. [47] (coverage = 5; GCS = 29), and by Fredriksen-Goldsen et al. [3] (coverage = 5; GCS = 669). The documents in this cluster focus on characterising the effect of minority stress in sexual minorities. The documents demonstrated that, as in other minority groups, sexual minorities experience poorer mental health as well as a higher risk of cardiovascular and metabolic diseases as compared to heterosexual individuals [3]. To explain health disparities in sexual and gender minorities, researchers attribute a major role to the experience of stigma perpetrated in social contexts and internalised biases [4]. Importantly, this cluster showed that sexual and gender minorities, who do not differ from majority populations in genetic, dietary, or other biological factors, nonetheless show comparable patterns of health vulnerability. This convergence across minority groups strengthens the argument that social determinants, including discrimination and minority stress, are key mechanisms underlying disparities in both mental and physical health.

### 4.9 Cluster #7: Racism and cardiovascular functioning

Cluster #7 emerged as the most recent cluster in the literature on autonomic nervous system functioning in minorities (mean year = 2011). Major citing documents included the ones authored by Brondolo et al. [48] (coverage = 10; GCS = 166), Agyemang et al. [49] (coverage = 9; GCS = 0), and Faconti et al. [50] (coverage = 9; GCS = 6). Studies in this cluster investigated ethnic and racial disparities in cardiovascular risk, as well as physiological responses to stress and racism (e.g., Refs. [8,44]). This cluster represents an important step towards understanding racism not just as a social phenomenon but as a measurable, physiologically consequential source of chronic stress that contributes to minority health disparities.

### 4.10 Cross-cluster synthesis and public health relevance

Taken together, the identified clusters show a progressive change in how the literature has conceptualised cardiovascular and autonomic functioning in minority populations. Earlier research largely focused on biological, genetic, dietary, and metabolic explanations of health disparities, whereas later clusters increasingly emphasised the role of social environments, racism, discrimination, stigma, and minority stress. This trajectory suggests that minority health disparities cannot be understood solely through individual-level or biological risk factors but should also be interpreted in relation to chronic exposure to unequal and stressful social conditions.

Mapping these themes is relevant for public health practitioners, clinicians, and policymakers because it clarifies where evidence has accumulated and where important gaps remain. For public health practice, the findings support the systematic assessment of discrimination-related stressors and structural barriers as contributors to cardiovascular risk. For clinical practice, they highlight the importance of considering minority stress and experiences of discrimination as contextual factors that may shape physiological vulnerability. For policy, the underrepresentation of sexual, gender, and neurodivergent minorities indicates the need for more inclusive surveillance systems, research funding priorities, and prevention strategies targeting populations that remain insufficiently studied.

### 4.11 Limitations

Some limitations should be acknowledged in the current study. First, the work relied on Scopus as the source database for retrieving the citing documents; therefore, relevant publications indexed only in other databases may not have been directly retrieved. However, this limitation was partially mitigated by the inclusion of the cited references from the retrieved records, which expanded the bibliographic base analysed in the document co-citation analysis and allowed the network to capture influential publications beyond the initial set of Scopus-indexed citing documents. Second, citation-based scientometric analyses tend to favour older and highly cited publications, which may reduce the visibility of recent studies and emerging topics. We partly mitigated this limitation by considering citation burstiness, which highlights publications receiving a rapid increase in attention over time. Relatedly, the present scientometric review was designed to map the structure and evolution of the literature, rather than to assess methodological quality, risk of bias, clinical validity, or intervention effectiveness. Therefore, the identified clusters should be interpreted as historically influential research domains rather than as evidence of clinical effectiveness or practice-ready interventions. Finally, the predominance of United States-based research should be interpreted as a characteristic of the literature mapped in this review rather than as a methodological limitation of the study itself. Nevertheless, this pattern may influence how minority status, racism, discrimination, and health disparities are conceptualised in the field, and highlights the need for future research from more geographically diverse contexts.

### 4.12 Conclusion

Health disparities are widely observed among minority groups and are often linked to chronic stress resulting from stigma and discrimination. Using a scientometric approach, the present study examined the literature on the physiological functioning of minority populations, identifying nine research themes across 1945 publications published between 1953 and 2024. The findings indicate that early research focused on biological and lifestyle explanations, such as genetics and diet, whereas more recent work has increasingly emphasised cardiovascular health disparities and the role of racism, discrimination, and minority stress.

From a public health perspective, these findings provide an evidence map that can inform research priorities, policy development, and practice. First, the shift from biological explanations towards social and structural determinants highlights the need to systematically assess discrimination, stigma, and minority stress as relevant contributors to cardiovascular and autonomic functioning. Second, the limited attention devoted to sexual, gender, and neurodivergent minorities indicates that future epidemiological and physiological studies should more consistently include these populations and collect minority-related variables. Third, the identification of racism and minority stress as emerging themes supports prevention strategies aimed not only at individual-level risk factors, but also at reducing chronic exposure to social stressors. For clinicians, the findings support greater attention to minority stress and discrimination as contextual factors that may contribute to cardiovascular risk and autonomic dysregulation. Although this scientometric review does not directly evaluate intervention effectiveness, it identifies areas where evidence is accumulating and gaps that are directly relevant for translating public health evidence into practice.

## Ethical statement

No ethical approval was required for this scientometric review. The study relied exclusively on previously published literature and publicly available bibliographic metadata. It did not involve human participants, animals, experimental procedures, clinical data, surveys, interviews, or the collection of identifiable personal information. As the analyses were performed on aggregated publication-level data, formal review by an ethics committee was not applicable.

## Author contributions

Conceptualisation: SF, AC, CO, RB, FL, GE; Formal Analysis: AC; Funding acquisition: CO, RB, FL, GE; Investigation: AC; Writing–original draft preparation: SF, AC; Methodology: AC; Writing–review and editing: SF, AC, CO, RB, FL, GE; Supervision: GE. All authors have read and agreed to the published version of the manuscript.

## Funding

This study was supported by PRIN 2022 (2022EL4MPH) funded by the Ministry of Education, University and Research, Italy. The funding source had no role in the study design, collection, analysis and interpretation of data, writing of the report and decision to submit the article for publication.

## Declaration of competing interest

The authors declare that they have no known competing financial interests or personal relationships that could have appeared to influence the work reported in this paper.
