# Supplementary material for: Research on cardiovascular functioning in minority groups: From biological explanations to social and discrimination-related determinants
Source: Public Health Pract (Oxf). 2026 Jul 8;12:100826. doi: 10.1016/j.puhip.2026.100826 (PMC13382016; doi:10.1016/j.puhip.2026.100826)
Supplement: Multimedia component 1 [file mmc1.pdf]

1 Bibliometric analysis on the citing documents

The preliminary bibliometric analysis was conducted to identify the most involved countries, the most productive authors, the most relevant journals, and the most common keywords in the literature about physiological functioning in minorities.

Based on the authors' affiliation strings, we computed bibliometric indicators describing countries' involvement in the field. From the results, the most involved countries are the United States of America (number of documents = 1109; frequency = 0.66; SCP = 1026; MCP = 83), the United Kingdom (number of documents = 152; frequency = 0.09; SCP = 114; MCP = 38), China (number of documents = 92; frequency = 0.06; SCP = 82; MCP = 10), and the Netherlands (number of documents = 69; frequency = 0.04; SCP = 53; MCP = 16). Figure 1 and Table 1 show the frequency with which individual countries appeared in the authors' list in publications related to the physiological functioning of minority groups. Additionally, Figure 2 shows that the strongest international collaborations involve the United States partnering with the United Kingdom, Canada, China, and the Netherlands.

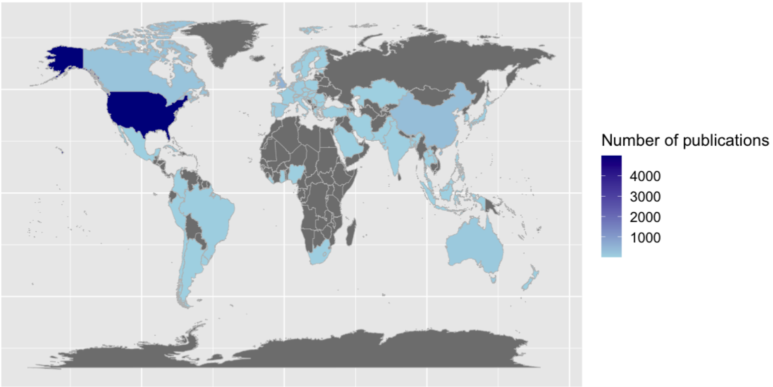

Fig. 1 World map illustrating the degree of each country's involvement in the literature on the physiological functioning of minority groups as measured by the number of publications. Publication frequencies are computed by taking into account all the authors contributing to the publications.

Item	Number of documents
**Countries**	
United States of America	1109
United Kingdom	152
China	92
Netherlands	69
Canada	44
**Authors**	
Agyemang C	36
Stronks K	22
Wang J	21
Caceres BA	20
Snijder MB	20
**Journals**	
Ethnicity and Disease	40
BMC Public Health	34
Journal of the American Heart Association	27
PLOS ONE	24
Journal of Racial and Ethnic Health Disparities	20
**Authors' keywords**	
Hypertension	196
Cardiovascular disease	165
Ethnicity	138
Health disparities	109
Diabetes	76

Table 1 Bibliometric analysis results showing the frequency of countries, authors, journals, and authors' keywords in the dataset. The analysis was conducted using the *bibliometrix* package for R (Aria and Cuccurullo 2017).

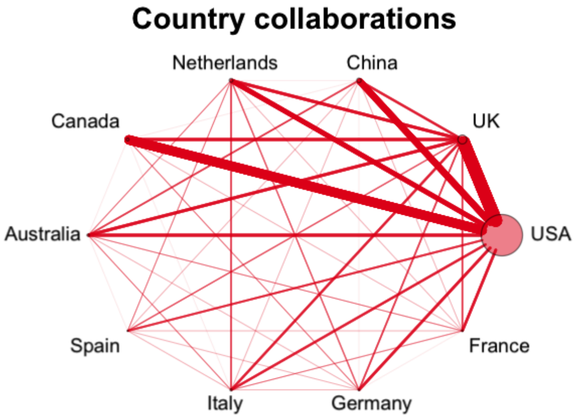

Fig. 2 Country collaboration network derived from co-authorship data. Each node represents a country appearing in the authors' affiliations, and edges denote co-authored publications between countries. Edge thickness reflects the strength of collaborative ties.

The most productive authors in the literature of interest are C Agyemang (number of documents = 36), K Stronks (number of documents = 22), and J Wang (number of documents = 21; see Table 1 for the list of the most productive authors in the field).

The most relevant journals for documents on physiological functioning in minorities are *Ethnicity and Disease* (number of documents = 40), *BMC Public Health* (number of documents = 34), the *Journal of the American Heart Association* (number of documents = 27), and *PLOS ONE* (number of documents = 24; see Table 1 for the list with the most relevant journals in the field).

The most common keywords are *hypertension* (number of documents = 196), *cardiovascular disease* (number of documents = 165), *ethnicity* (number of documents = 138), *health disparities* (number of documents = 109), *diabetes* (number of documents = 76), *obesity* (number of documents = 71), *racism* (number of documents = 70), *risk factors* (number of documents = 69), *blood pressure* (number of documents = 66), and *race* (number of documents = 66; see Table 1 for the list with the most common keywords in the field). Figure 3 depicts the co-occurrence patterns between the 50 most occurring keywords.

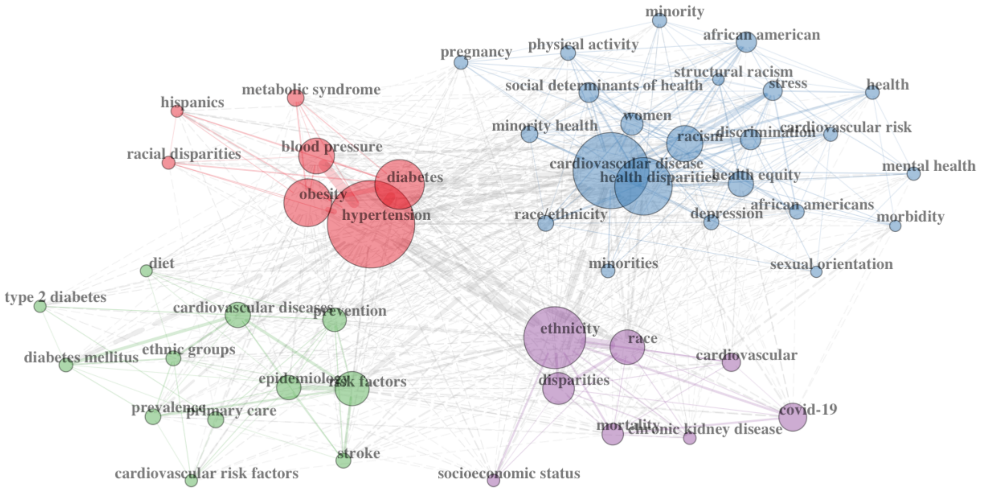

Fig. 3 Top 50 keywords co-occurrence patterns.

References

Aria M, Cuccurullo C. bibliometrix: An R-tool for comprehensive science mapping analysis. Journal of informetrics. 2017;11(4):959–975.
